# Current Topics in OCT Applications in Vitreoretinal Surgery

**DOI:** 10.3390/bioengineering12090962

**Published:** 2025-09-07

**Authors:** Shintaro Horie, Takeshi Yoshida, Kyoko Ohno-Matsui

**Affiliations:** 1 Department of Ophthalmology and Visual Science, Institute of Science Tokyo, Tokyo 113-8519, Japan; 2 Department of Ophthalmology and Visual Science, Department of Advanced Ophthalmic Imaging, Institute of Science Tokyo, Tokyo 113-8519, Japan

**Keywords:** optical coherence tomography, vitreoretinal surgery, macular holes, retinal detachment, epiretinal membrane

## Abstract

Optical coherence tomography (OCT) is an indispensable tool in modern ophthalmology, where it is used in prior examinations, among various instruments, to assess macular or vitreoretinal diseases. Pathological macular/retinal conditions are almost always examined and evaluated with OCT before and after treatment. Vitreoretinal surgery is one of the most effective treatment options for vitreoretinal diseases. OCT data collected during the treatment of these diseases has accumulated, leading to the reporting of a variety of novel biomarkers and valuable findings related to OCT usage. Recent substantial developments in technology have brought ultra-high-resolution spectral domain/swept source OCT, ultra-widefield OCT, and OCT angiography into the retinal clinic. Here, we review the basic development of the instrument and general applications of OCT in ophthalmology. Subsequently, we provide up-to-date OCT topics based on observations in vitreoretinal surgery.

## 1. Introduction

The introduction of optical coherence tomography (OCT) represents one of the most remarkable breakthroughs in retinology and ophthalmology. OCT images provide detailed internal structural information about the retina in real time [1]. In the 1990s, OCT technology began to be applied to ocular imaging [2], and the first commercial OCT instrument was produced by Carl Zeiss Inc. (Thornwood, NY, USA) in 1996.

First-generation OCT is based on time domain technology [3]. Spectral domain (SD), Fourier domain (FD), and swept source (SS) technology have realized OCT images with higher resolutions, and deeper and wider scales [4,5,6,7]. Recent commercial models of swept source OCT or SD-OCT for ocular fundus can show 10 layers of histological sections with high resolution. For instance, retinal hemorrhages or hard exudates derived from the breakdown of the inner and outer retinal barrier can be precisely located as being in the outer plexiform layer, inner nuclear layer, subretinal space, etc. Examining the membrane or layers of the vitreoretinal interface is another significant target of OCT imaging. Many retinal diseases develop a complex vitreoretinal pathology, and these layers are potential targets for surgical treatment.

Pars plana vitrectomy is the most sophisticated reliable method of vitreoretinal surgery. Closed vitrectomy dates back to the 1970s, when it began to be indicated for non-clearing vitreous hemorrhage [8,9]. The indications have been gradually extended across several vitreoretinal problems such as macular diseases, retinal detachment, proliferative retinopathy, and variable vitreoretinal conditions. This progress is owing to successive improvements in vitrectomy machines and optical microscope viewing systems, and numerous technical inventions [10]. The spread of vitrectomy in clinical use has necessitated more detailed observations of the macula and retina by OCT, and OCT has become an essential tool in making decisions for surgery and follow-up assessment. For example, vitreous tractional forces occurring at the interface of the retina can cause retinal detachment, retinoschisis, or retinal edema [11]. Such unfavorable vitreous traction on the retina is a primary target for removal by vitreous surgery. In addition, OCT can be used to preoperatively determine the locations and sizes of subretinal hemorrhages or fluids that need to be removed by surgery.

In this paper, we explore the footprints and general applications of OCT in major vitreoretinal disorders. Subsequently, this review provides an update on various topics of OCT-based studies in vitreoretinal surgery. Although OCT technology is used to observe anterior segments of the eye such as the cornea and anterior chamber, or to determine the iridocorneal angle, these subjects are not addressed in this review.

## 2. Overview of OCT Technology in Retinal Imaging

In the 1990s, OCT technology was introduced for the imaging of retinal diseases [2]. This was a real breakthrough in ophthalmology, and the first commercial OCT instrument produced by Carl Zeiss Inc. spread rapidly across retinal clinics worldwide. Previously, it had not been possible to obtain real-time information about the inner structure of the retina. OCT imaging allowed for the locations, sizes, or shapes of pathological findings in the posterior ocular segment to be identified with high accuracy. The basal technology of OCT has evolved from time domain technology. Following the early model, second-generation instruments were developed, which adopted spectral domain (SD) or Fourier domain (FD) technology. Subsequently, swept source technology [12] was developed in more recent models, with ultra-high-resolution and wider- or deeper-range OCT images becoming feasible [4,5,6,7]. For instance, modern swept source OCT can clearly illustrate 10 layers of the inner retina similarly to histological sections [13]. Hard exudates derived from the breakdown of the blood–retinal barrier can be distinguished in their exact locations, such as the outer plexiform layer, inner nuclear layer, or subretinal space, which is impossible with fundus photography,

There has been continuous exploration of how to obtain higher-resolution OCT images in shorter times as the instrument has been developed. Additionally, the area captured in a single OCT map has been expanded. Namely, the acquisition of images with wider and deeper fields has been investigated. An example of the former is found in a recent swept source OCT device (OCT-S1, Canon, Hong Kong, China), which covers a wide field of the retina (maximum 21 × 23 mm) up to the periphery [14]. Enhanced depth (ED) imaging technology has emerged from later progression, which enables us to observe the whole range of the choroidal layer [15]. Furthermore, scleral fibers have been targeted and visualized by polarization-sensitive OCT. With this method, the characteristics of scleral fiber patterns in pathologic myopia were determined [16,17].

Retinal vascular flow is detected using OCT technology, and this novel imaging technology is termed OCT angiography (OCTA). A retinal image obtained by OCTA displays the vascular network of the retina in detail and part of the choroid [18,19,20]. This is not a substitute for conventional retinal and choroidal angiography with an infusion dye such as fluorescein or indocyanine green. It is a completely innovative method because it can visualize the vascular network across different layers of the posterior segment in three dimensions. For example, the superficial or deep capillary plexus of microaneurysms of diabetic retinopathy can be identified individually, which is impossible in conventional fluorescein angiography. Furthermore, to detect and measure retinal vascular flow and volume, Doppler OCT (DOCT) has been developed [21]. Spontaneous retinal venous pulsation was measured quantitatively using DOCT or intensity-based OCT, implicating the progress in hardware and software solutions [22]. Operating microscopes are additional novel types of OCT instruments (intraoperative OCT), well known for their application in MH surgery [23] and retinal detachment surgery [24]. A handheld model has also recently been developed. This is used in neonatal intensive care units in the management of retinopathy of prematurity [25].

**Figure 1:**
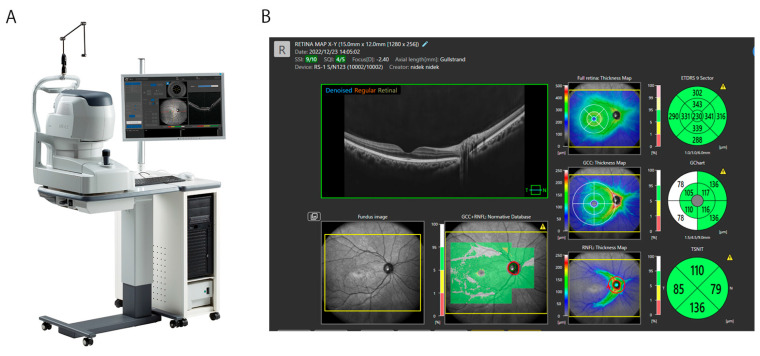
(**A**): The latest commercial model of an OCT instrument, *Glauvas*^®^ (NIDEK Inc. Gamagori, Japan), for retinal examination, with a 250,000 A-scan speed. The dimensions of the captured map image are 16.5 mm × 15.0 mm and 4.2 mm in depth. (**B**): A retinal map of the right eye in a healthy control. A retinal tomographic image with full thickness, a ganglion cell complex, and a retinal nerve fiber layer is shown.

Recent commercially available models of OCT instruments can obtain images in very short times, decreasing the burdens on both the patient and the examiner. For example, 12 radial line (tomographic) sections or a retinal map can be obtained within a few seconds. Developments have pursued images with higher resolutions and expanded widths and depths (Figure 1). Obtaining a wider area up to the equatorial retina is valuable because vitreoretinal lesions can exist throughout retinal surfaces beyond the macula area. Observing the vitreous space anterior to the retinal surface is also useful because intravitreous lesions are a target of vitrectomy. For these reasons, the technical progress in OCT instruments has been steady and has continuously profited vitreoretinal surgeons. However, it is still challenging to obtain clear images in cases of media opacity such as severe cataracts, vitreous opacity, or vitreous hemorrhages [26,27].

## 3. Clinical Applications of OCT in Vitreoretinal Diseases

### 3.1. Macular Diseases

The macula is located at the central bottom of the ocular fundus. It is the most functionally essential part of the retina, providing our vision. Decreased visual acuity, metamorphopsia, or even scotoma can exist in a patient with macular disorders. Thus, examining the macular condition by OCT takes priority in a clinical respect. Here, we describe examples of OCT’s application for representative macular diseases.

**Figure 2:**
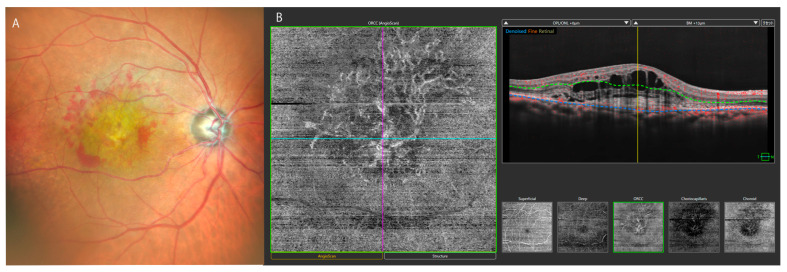
Fundus image of right eye and OCT angiography/OCT images of age-related macular degeneration (type 2) in 81-year-old woman. (**A**): SLO color image of *Mirante* (NIDEK); (**B**): OCTA/OCT of *Glauvas* (NIDEK) image of type 2 macular neovascularization (MNV).

*Age-related macular degeneration (AMD)* is one of the leading severe vision-threatening diseases in elderly people worldwide, especially in Western countries. Because it usually causes severe visual impairment in the central visual field, its management is critical. Not only age but also smoking and consuming a high-saturated-fat diet are considered major risk factors for these diseases [28]. Classically, angiography with fluorescein and indocyanine green was a major tool for detecting macular neovascularization (MNV). Exudative fluid from the MNV of wet AMD is shown as dye leakage in fluorescein angiography. However, OCT enables us to determine the location and volume of a hemorrhage or fluid [29,30]. Subsequently, OCTA technology has the potential to substitute classical angiography in diagnosis and evaluation. Because OCTA is noninvasive in terms of the potential for anaphylaxis, it has become preferred over fluorescein/indocyanine green angiography, substituting the latter. Thus, OCTA has become ever more indispensable not only in wet AMD [31] but also in other types of MNV such as myopic macular degeneration [32] and polypoidal choroidal vasculopathy [33] (Figure 2).

*Macular hole (MH)* is a pathological condition of a hole-shaped defect in the macula. It causes visual impairment by decreasing visual acuity or causing metamorphopsia, etc. The severity of its symptoms usually depends on the duration from the onset and the size of the hole. MH, subclassified into idiopathic MH and secondary MH, is one of the best examples of a disease that can be diagnosed using OCT [34]. Initially, J.D. Gass reported the classification of idiopathic MH by stage based on clinical observation through slit lamp ophthalmoscopy [35]. More recently, a new classification scheme based on OCT findings were reported by the international vitreomacular traction study group [36]. Vitreoretinal surgery is the first-line choice in the treatment of MH. The accurate determination of stage and size is critical for the success of surgery.

*Epiretinal membrane (ERM)* is another typical target of vitreoretinal surgery [37]. The clinical definition of “epiretinal membrane” usually includes idiopathic epiretinal membrane and various epiretinal complications secondary to ocular inflammation, diabetic retinopathy, rhegmatogenous retinal detachment, or even taut posterior vitreous face. Because it is one of the best indications of OCT imaging, novel insights have been accumulated based on the observations.

### 3.2. Retinal Detachment

*Retinal detachment* is defined as separation between the photoreceptor layer and retinal pigment epithelium (RPE). Rhegmatogenous retinal detachment (RRD) is a major form of retinal detachment. RRD is caused by vitreous traction and retinal defects such as retinal tears or holes [38]. Other forms of retinal detachment include tractional retinal detachment (TRD) and serous retinal detachment, and their underlying mechanisms differ from those of RRD. Because visual acuity is primarily dependent on macula/fovea status, the presence and amount of submacular/foveal fluid are critical. Whether RRD is accompanied by macular detachment is vital because this potentially determines the recovery of vision following surgery. Therefore, OCT is a powerful tool for RRD [39], enabling the detection of even small amounts of subretinal fluid in the macular area. If the macula/fovea is detached, the RRD is classed as “macula off”, while “macula on” denotes a better status with macular attachment. OCT is also a powerful tool for detecting tiny retinal holes if the retinal break is in the macular area in high-myopic eyes [40]. *Retinoschisis* (RS) is a similar condition of retinal detachment, although it does not involve separation between the photoreceptor layer and RPE but involves intraretinal rupture, usually in the outer plexiform layer [41]. Myopic tractional maculopathy is a severe retinal condition leading to visual loss and blindness in patients with high myopia [42]. This disorder is frequently accompanied by macular RS, MH, and/or RD. Whether the disease has progressed from macular RS to retinal RD is critical in its management and the decision regarding surgery [42]. Careful observation with OCT is indispensable for this purpose.

### 3.3. Diabetic Retinopathy

**Figure 3:**
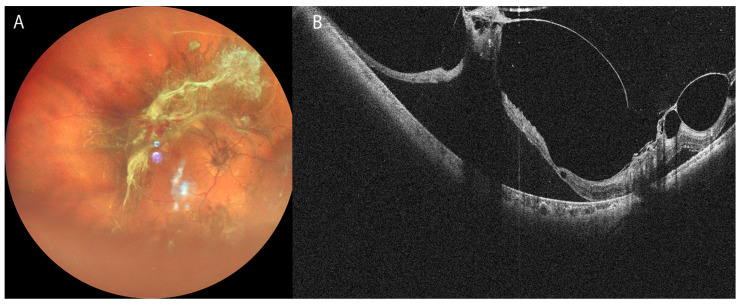
Ultra-widefield (UWF) fundus and OCT images of severe proliferative diabetic retinopathy in 51-year-old man. (**A**): UWF image of SLO (Mirante); (**B**): UWF OCT (Canon S1) topographic image of A. Macula-off tractional retinal detachment caused by vitreous traction of fibrovascular tissue and posterior hyaloid.

Diabetic retinopathy (DR) is one of the major vision-threatening diseases worldwide. As the diet and lifestyle of Western countries have been popularized across the world, the prevalence of diabetes mellitus (DM) has increased up to the levels observed in developed countries [43]. DR is usually classified into several categories based on the presence of neovascularization. Non-proliferative DR (NPDR) is classified into three categories—mild, moderate, or severe—while proliferative DR (PDR) is not subclassified [44]. Currently, diabetic ME (DME) is usually assessed and treated based on OCT findings [45]. The distribution or volume of extracellular fluid derived from the breakdown of the blood–retinal barrier is easily assessed using topographic OCT images. Numerous biomarkers predicting the prognosis of the diseases have been reported [46], and the significance of multimodal assessment was proposed [47,48]. OCTA is also useful in DME management as well as in MNV. Microaneurysms (MAs) are the earliest and most frequent finding pertaining to DR. MAs are considered a major cause of intraocular exudative fluid leading to macular edema. OCTA is the best method for detecting MAs because it can scan different layers of the retina [18,19]. OCTA is superior to fluorescein angiography (FA) because it can distinguish MAs of the inner microcapillary plexus from those of the outer microcapillary plexus. It is superior to FA because the latter presents a risk of anaphylaxis caused by fluorescein infusion. Thus, OCTA has become more popular for the management of the disease [49]. PDR is also a potentially good target of OCT observation [50]. In PDR, the fibrovascular membrane is frequently distributed across a wider area than that in idiopathic ERM. Because previous models of OCT instruments could only capture a limited area of the retina surrounding the macula, extensive areas of fibrovascular tissue had not been entirely captured. However, several recent models of widefield OCT instruments can overcome this issue if the vitreous hemorrhage is less than moderate (Figure 3).

## 4. Contemporary Topics in OCT-Based Assessment in Vitreoretinal Surgery

### 4.1. Macular Hole (MH)

#### 4.1.1. Novel Biomarkers of MH in Vitreoretinal Surgery

Several OCT biomarkers have been proposed for predicting the prognosis of MH following surgery. For example, Guerin et al. reported that the *external limiting membrane (ELM) aperture* was a reliable prognostic factor for postoperative anatomical and functional outcomes in full-thickness MH (FTMH) surgery [51]. The novel biomarker of the *photoreceptor integrity index*, which is calculated by OCT measurement, was proposed for evaluating photoreceptor loss in MH, and postoperative functional and anatomical recovery [52]. *Epiretinal proliferation (EP)* accompanied by FTMH was noted. The presence of EP in FTMH may indicate worse visual recovery than that without EP. EP can be accurately identified using en face OCT even when small [53]. In lamellar MH, which is distinguished from FTMH, the *retinal layer continuity at the lamellar MH and MH height* observed by OCT have the potential to be good preoperative markers predicting postoperative VA [54]. *Concentric macular dark spots* found in SD-OCT indicated a dissociated optic nerve fiber layer (DONFL) following peeling of the internal limiting membrane (ILM) in idiopathic MH (iMH) [55]. Additionally, novel morphologic stages according to SD-OCT were proposed, and the prognosis of each stage was examined [56].

#### 4.1.2. Advanced Surgical Techniques of MH and OCT (Table 1)

Large FTMHs (usually defined as those with sizes > 400 μm) are relatively difficult to treat with conventional ILM peeling alone and gas tamponade. The *ILM flap* technique and *ILM insertion* to MH are the most typical solutions for treating large FTMH. There have been many studies comparing these techniques. For instance, it is reported that both techniques were associated with high rates of MH closure in large MHs, and the inverted ILM flap technique seemed to be more effective than ILM insertion in improving visual outcomes [57]. To date, the ILM flap technique has been the main target of investigation. A uniquely shaped non-inverted single-layer “plastic bag” ILM flap technique was reported as a novel effective method for large MHs [58]. Another novel surgical treatment for large MH combined an inverted ILM flap and the subretinal injection of a balanced salt solution in the FTMH. In this study, extending the detachment led to successful closure of a chronic FTMH [59]. In patients with coexisting RRD and FTMH cases, the inverted ILM flap technique led to significant improvements in anatomical and visual outcomes [60]. Compared to standard ILM peeling or fovea-sparing ILM peeling, the inverted ILM flap technique resulted in similar VA, but there is relatively little evidence of postoperative FTMH in the treatment of lamellar macular holes in myopic tractional maculopathy [61]. There is a study proposing a modified petaloid technique for FTMH and analyzing the impacts of the preoperative shape of MHs on postoperative visual acuity [62]. In the case of noncausative FTMHs accompanied by RRD, the inverted ILM flap technique with air/gas tamponade resulted in favorable anatomical and functional outcomes for FTMHs [63]. In eyes with extremely high myopia, an inverted ILM flap achieved better visual outcomes than ILM insertion [64]. Upon comparing the inverted ILM flap technique and conventional peeling, postoperative metamorphosia was not found to significantly differ between the two groups [65]. For large MHs closed following surgery, both the ILM-peeling- and ILM-insertion-treated groups showed a significantly improved microstructure and microperimeter in the fovea, but the ILM-insertion-treated group showed less efficient recovery [66]. The novel method of peeled ILM reposition has advantages over conventional ILM peeling in that it leads to better microstructural outcomes for the inner retina and functional recovery [67]. In lamellar hole-associated epiretinal proliferation, the embedding technique improved visual acuity more than conventional ILM peeling and yielded better anatomic outcomes with no FTMH formation [68]. *Amniotic membrane transplantation* is another major technique for closing large MHs [69,70,71,72,73]. This is a similar strategy for refractory MHs using autologous patches such as *retinal transplants* [74,75], *autologous platelet-rich plasma plugs* [76], or *platelet-rich plasma* for lamellar macular holes [77]. Another approach, the *hemitemporal ILM peeling* method, has been reported. In this method, the retinal migration [78] or DONFL score [79] for complications after surgery was analyzed. The requirement for ILM peeling with or without an ILM flap for small idiopathic MHs, contrary to large recurrent MHs, was also investigated. In these studies, the conventional method was reported to be sufficient [80,81]. Furthermore, an interesting surgical attempt and observation of the *intraoperative closure of large FTMHs* were recently reported. In this study, FTMHs were successfully closed during surgery under a PFCL bubble with the passive extrusion of fluid [82].

**Table 1:** Technical topics in vitrectomy for macular holes.

Technique	Indications	Characteristics	References
Inverted ILM flap	Large FTMHs, lamellar ILMHs	High closure rate, functionally good outcome	[57,59,60,61,62,63,64,65]
ILM insertion	Large FTMHs, lamellar ILMHs	High closure rate	[57,64]
Amniotic membrane transplantation, autologous tissue patch	Large FTMHs	High closure rate, requiring equipment	[69,70,71,72,73,74,75,76,77]
Hemitemporal ILM peeling	FTMHs	Reducing damage of retinal nerve fiber layer	[78,79]
Intraoperative closure with PFCL	FTMHs	Original method	[82]

ILM, internal limiting membrane; FTMHs, full-thickness macular holes; PFCL, perfluorocarbon liquid.

#### 4.1.3. More Insights into MH and OCT

The use of *intraoperative OCT* for the vitrectomy of MHs is one of the best applications of this new instrument. This surgical option is usually performed with a three-dimensional digital viewing microscope [83], which enables intraoperative vitreoretinal observation. For instance, it enabled the characteristics of ILM forceps in peeling to be evaluated [84]. *OCTA* is also applied to examine the postoperative foveal avascular zone (FAZ), and a study reported that no differences in microvascular parameters were identified pre- and post-surgery [85,86]. Among the various types of OCT, en face OCT with two-dimensional images was found to be practical for distinguishing the closure patterns of MHs during postoperative periods [87]. Preretinal abnormal tissue proliferation in MHs was also well demonstrated by en face OCT [88]. In addition, choroidal findings in MH surgery were reported. The *choroidal hypertransmission width* [89], *choroidal thickness* of an iMH and idiopathic ERM (iERM) [90], and *choriocapillaris disruption in iMHs* were examined in these studies [91].

### 4.2. Epiretinal Membrane (ERM)

#### 4.2.1. Biomarkers of ERM in Vitreoretinal Surgery

The *ectopic inner foveal layer (EIFL)* is considered a significant biomarker in ERM surgery. EIFL thickness has emerged as a critical determinant of postoperative metamorphopsia and predictor of functional outcomes after ERM surgery [92]. After the epiretinal membrane was peeled, the EIFL thickness was reduced, and the normal foveal pit structure was restored with visual acuity improvement [93]. In another study, the EIFL was not a predictor for the development of distance-corrected visual acuity 1 year after surgery in terms of the central macular thickness [94]. In addition, preoperative *ellipsoid zone (EZ) disruption* and *retinal nerve fiber layer schisis* on OCT may identify patients who are more likely to have meaningful improvements in postoperative visual outcomes for idiopathic ERM [95]. Using artificial intelligence (AI), the quantification of OCT biomarkers in ERM surgery was investigated, and the *EZ and external limiting membrane (ELM) integrities* remained crucial prognostic factors, emphasizing the importance of preoperative analysis [96]. ERMs with severe inner retinal structural changes are associated with a sticker ILM, and peeling the ERM led to a DONFL [97]. Additionally, *the ERM-ILM distance* was investigated and eyes with larger distances were likely to show preserved ILMs during ERM surgery, potentially minimizing neuroretinal damage in eyes with glaucoma and related diseases [98]. Upon examining morphologic macular changes following surgery, OCT showed that the fovea moved nasally, and the distance between the superior and inferior vascular arcades increased significantly. However, such changes were relevant to the improvement in BCVA but not metamorphopsia [99]. The presence of *disorganization of retinal inner layer (DRIL)* before and after surgery, *microcysts, and outer retinal change* after surgery were related to worse visual outcomes [100]. iERM patients with DRIL have more intraoperative adverse events and limited benefits from surgery, which should be considered in the decision whether to perform membrane peeling [101]. In the morphological aspect, *foveal hernitation* in iERM leads to a unique macular appearance. Eyes with foveal herniation in iERM experienced various visual disturbances based on the involvement of the inner retinal layers [102]. In other studies, different types of ERM–foveoschisis were examined and associated with visual function following surgery [103,104] (Table 2).

#### 4.2.2. Novel Topics in ERM Surgery and OCT

The novel technique combining *vitrectomy with subretinal BSS injection* has demonstrated significant visual and anatomical efficacy in treating severe iERM and can rapidly enhance BCVA and downgrade the EIFL stage [109]. *AI technology* is also useful in ERM management; it is reported that AI outperformed human grading in detecting ILM removal from OCT scans [110]. The deep learning model ResNet101 achieved high overall performance in predicting postoperative VA from preoperative OCT images [111]. *OCTA* was also useful in detecting ERM-induced changes in the superficial capillary plexus (SCP) [105]. OCTA findings reveal that both increased radial peripapillary capillary (RPC) density and reduced SCP density at baseline could serve as predictors of better visual outcomes after surgery [106]. A classification based on the FAZ area of OCTA can reflect the progression of iERM and is helpful for the postoperative prognosis [107]. The RPC density significantly decreased after the surgical removal of ERM, and ILM peeling can cause peripapillary microvascular damage starting in the inferior sector [108]. Preoperative choriocapillaris perfusion analyzed by OCTA is a biomarker for poor functional and anatomical prognosis after surgery in iERM [112]. EDI-OCT is also used in ERM cases, and the *choroidal thickness and choroidal vascular index* (CVI) were evaluated in the postoperative period [113]. The CVI was significantly correlated with visual outcomes after surgery, representing a potential predictive marker of iERM [114]. Vascular flow indices were monitored, and the relative difference in vascular flow provided objective estimates of changes due to surgery [115]. The maximum depth of the retinal folds with the parafovea measured using OCT is significantly correlated with the macular ERG amplitude and alpha-smooth muscle actin expression in the specimen obtained in surgery [116]. In addition, *adaptive optics OCT* revealed discernible differences in the foveal cones of eyes with ERM compared to control eyes that were closely related to visual function impairments [117]. *Postoperative microcystoid macular edema* was a frequent finding in ERM and a rare occurrence in FTMH, suggesting that it is related to Müller cell disruption and iatrogenic damage [118]. A study reported difficulty in distinguishing iERM and secondary ERM using OCT [119]. Another study reported that the recurrence of ERM is generally preceded by the *persistence of ERM fragments* found in the early postoperative period. The growth of ERM persistence from the parafoveal region was often the origin of foveal ERM recurrence [120]. Moreover, there is a study reporting a *substantial enhancement in stereopsis following iERM surgery*, with both interocular and monocular OCT parameters independently influencing postoperative stereopsis [121].

### 4.3. Rhegmatogenous Retinal Detachment (RRD)

#### 4.3.1. Morphologic Stages of RRD and Retinal Displacement Following Surgery

A sequential *morphologic staging* system for RRD based on OCT was recently proposed. Outer retinal changes are graded into five stages, from the earliest stage of the separation of the neurosensory retina from the retinal pigment epithelium to the severe stage of the complete loss of inner or outer retinal segments [122]. An increased morphologic stage is associated with delayed recovery of the outer retinal bands and faster development of the epiretinal membrane after RRD repair [123]. It is reported that the early morphologic stages may benefit from more urgent intervention [124]. *Retinal displacement* is one of the most annoying complications associated with RRD surgery, especially in macula-off RD. It causes visual impairment such as decreased visual acuity, metamorphosia, or aniseikonia [125]. It was found to be more prevalent following pars plana vitrectomy surgery than scleral buckling [126]. Retinal displacement was identified by postoperative foveal location using photographic images, and OCT could explain incomplete visual recovery by demonstrating foveal misalignment or changes in foveal microstructures such as disorganized retinal outer layers [125]. There is also a study reporting the effects of the peeling of peripheral vitreous cortex remnants on postoperative retinal displacement [127].

#### 4.3.2. Biomarkers of RRD and Vitreoretinal Surgery (Table 3)

Many practical biomarkers related to prognosis in retinal detachment surgery have been reported, and notable findings in treatment have been accumulated. For example, the *ellipsoid zone (EZ) and external limiting membrane (ELM) reflectivity* as determined through quantitative assessment were significantly associated with better postoperative visual acuity (VA) in RRD patients [128]. Similarly, reduced and variable *inner segment/outer segment junction (IS/OS) band reflectivity* on OCT images was found to be associated with reduced macular sensitivity [129] or multifocal-ERG amplitude [130]. Other novel markers such as the *height of retinal detachment at the fovea*, the *disruption of the EZ and/or ELM*, the *presence of intraretinal cystic cavities (ICCs)*, or fovea detachment were associated with poor postoperative VA [39]. The presence of *hyperreflective dots (HRDs) in the outer retina* was significantly associated with the morphologic stage, extent, duration, and height of the RRD before surgery. In addition, reduced VA and cystoid macular edema following surgery were also related to HRDs [131]. Similarly, *subretinal hyperreflective points (HRPs)* indicated an inflammatory response and were a negative predictor for postoperative visual recovery [132]. *Preretinal HRDs* observed in OCT were found to be associated with the occurrence of ERM following vitrectomy for RRD [133]. Similar findings of a *preretinal hyperreflective layer* and saw tooth of the retinal surface in OCT were associated with intraoperative vitreous cortex remnants (VCRs), making them potential preoperative markers [134]. There are also studies reporting OCT findings such as multiple subretinal particles [135] and outer retinal folds following pars plana vitrectomy for RRD [136]. Early postoperative retinal displacement and *discontinuity of the external limiting membrane/interdigitation zone* in postoperative OCT were found to be potential predictors of postoperative visual acuity [125]. Additionally, preoperative choroidal hypertransmission with preserved foveal EZ and RPE was indicated to be a predictor of surgical failure in myopic foveoschisis [137].

**Table 3:** Biomarkers of OCT in retinal detachment surgery.

Biomarkers	Major outcomes	References
EZ/ELM, IS/OS reflectivity	Postoperative VA, macular sensitivity, multifocal-ERG amplitude	[128,129,130]
HRDs in outer retina	Preoperative morphologic stage, postoperative VA, CME	[131]
Epiretinal HRDs or layers	Epiretinal membrane, VCRs	[133,134]
Subretinal HRPs, multiple SRPs, postoperative outer retinal folds, retinal displacement, discontinuity of ELM	Postoperative reduced BCVA	[125,132,135,136]
Discontinuity of interdigitation zone	Metamorphosia	[125]

EZ/ELM, ellipsoid zone/external limiting membrane; IS/OS, inner segment/outer segment; ERG, electroretinogram; HRDs; hyperreflective dots; HRPs, hyperreflective points; CME, cystoid macular edema; VCRs, vitreous corte remnants; SRPs, subretinal particles; BCVA, best corrected visual acuity.

#### 4.3.3. Current Additional Topics in RRD and OCT

Attempts to use *deep learning* and *artificial intelligence (AI)* in RRD management have been made. Through this technology, an increased outer nuclear layer and photoreceptor and retinal pigment epithelium thickness measured by OCT were found to be associated with better postoperative visual outcomes in macula-off retinal detachment [138]. AI could predict functional outcomes [139] or anatomical outcomes [140] after RRD surgery. A method of patching for retinal tears, similar to methods for FTMH, was reported. In that study, the *amniotic membrane* was adequately adapted to retinal breaks in RRD, which was confirmed by SD-OCT [141]. Regarding the retinal circulation, the use of *OCTA* in RRD cases was reported. It revealed microvascular changes in patients with RRD vitrectomy. A difference in vascularity was observed in RRD eyes compared to unaffected fellow eyes, while no difference was seen in a different gas tamponade group [142]. Additionally, there is a new proposal of categorizing the fovea involvement in RRD as fovea-on, fovea-off, and fovea-split based on OCT findings [143]. Further topics include the *microstructural change following ILM peeling* in RRD surgery [144] and the *choroidal thickness* examined during RRD surgery [145]. Differences in choroidal vascular status between different tamponades of silicone oil (SO) or gas were also investigated [146].

### 4.4. Miscellaneous Topics in OCT and Vitreoretinal Surgery

OCT has revealed novel insights into not only macular disorders but also variable retinal diseases. In this section, several recent reports yielded by OCT-based studies are supplemented. Cases of *peripapillary retinoschisis* without a visible optic disc pit but with abnormal lamina cribrosa defects in OCT demonstrated improved BCVA and central foveal thickness following vitrectomy [147]. An *optic disc pit* was functionally and anatomically improved by vitrectomy with an autologous platelet concentrate patch, as evaluated using OCT [148]. OCT findings associated with *SO tamponade* are another hot topic in this field. The influences of SO tamponade on BCVA [149], RNFL thickness, and cystoid macular edema (CME) were studied. New-onset CME following SO tamponade may affect the inner nuclear layer initially, followed by outer nuclear layer involvement, and resolution was observed after the removal of SO [150]. Prolonged SO tamponade was a significant predictor of the development of cystoid macular edema (CME) [151]. Additionally, thinning of the macula after the repair of macula-on RRD with SO tamponade was observed, as was recovery following SO removal [152]. Interestingly, the incidence of pseudophakic CME as detected using OCT was not different between eyes with prior vitrectomy with gas tamponade versus SO tamponade [152]. *Widefield OCT* was stated to be useful in examining preoperative posterior vitreous detachment in ERM or MH cases [153]. In addition, the use of intraoperative OCT to deliver subretinal tPA in vitrectomy was reported to be safe and efficacious [154]. Operative digital enhancement of the macular pigment during macular surgery combined with *intraoperative OCT* was also recommended [155]. In recent studies of deeper tissue observed using OCT, the *choroidal vascularity* index as measured by EDI OCT was found to be decreased after surgery for *myopic traction maculopathy* [156]. In addition, a new attempt to classify and grade *myopic maculopathy* (ATN) based on OCT characteristics was proposed [41]. In terms of *DR*, the OCT-based assessment of DME following PDR surgery was reported [157]. In an OCTA study, postoperative BCVA following PDR surgery was related to OCTA parameters of both the superficial and deep capillary plexus and choriocapillaris plexus [158]. In cases of DME, superficial FAZ as measured by OCTA was decreased to a higher extent after vitrectomy with ILM peeling or intravitreal anti-VEGF injection [159].

## 5. Discussion and Conclusions

We review the basic development of the instrument and general applications of OCT in ophthalmology. Subsequently, we provide an update on recent topics related to OCT based on observations in vitreoretinal surgery. Since the development of OCT technology, its clinical significance has steadily increased year after year [1]. Recent clinical applications of OCT in macular diseases, retinal detachment, and diabetic retinopathy are overviewed in the third section. Subsequently, topics in OCT-based assessment in vitreoretinal surgery in the past few years are surveyed and summarized. OCT marked a true breakthrough in retinal imaging, offering ophthalmologists a wealth of new insights into macular and retinal disorders [1,160]. Prior to the advent of OCT/OCTA, evaluation of the macula relied heavily on fundus ophthalmoscopy or conventional angiography, the latter carrying a potential risk of anaphylaxis [161]. These conventional methods are still useful but lack much essential topographic information about the retina. Today, we can more precisely identify the pathological changes in the posterior eye segment with OCT, in size, form, or density [162]. The resolution, width, and depth of the images have been continuously improved. The recent advent of a portable handheld OCT model [25] provides great potential for healthcare systems worldwide. However, it is still challenging to obtain clear images with it in cases of media opacity such as severe cataracts, vitreous opacity, or vitreous hemorrhages [26,27].

When a vitreoretinal surgeon plans a surgery for any vitreoretinal disease, OCT images are valuable and also utilized in the follow-up period after surgery [163]. Intraoperative OCT is an additional powerful tool [23]. This review provides an update on various current OCT topics related to vitreoretinal surgery. Although it only includes recent studies, the obtained information is huge and indicates great findings. However, the progress in OCT instruments is ongoing, and ascertaining what has been improved and what remains imperfect in OCT images is essential for future progress. With the further development of OCT technology and vitreoretinal surgery, we will certainly become ever closer to knowing the real nature of retinal diseases.

## Figures and Tables

**Table 2:** OCT biomarkers in epiretinal membrane surgery.

Techniques	Major outcomes	References
EIFL thickness	Postoperative VA and metamorphopsia	[92,93,94]
EZ disruption, EZ-ELM integrity, RNFL schisis	Poor postoperative VA improvement	[95,96,97]
DRIL	Poor visual outcome, limited surgical outcome	[100,101]
Foveal herniation	Variable visual disturbances	[102]
RPC/SCP density, FAZ (OCTA)	Postoperative VA	[105,106,107,108]

EIFL, ectopic inner foveal layer; EZ, ellipsoid zone; RNFL, retinal nerve fiber layer; ELM, external limiting membrane; DRIL, disorganization of retinal inner layer.

## Abbreviations

The following abbreviations are used in this manuscript:

OCT	Optical coherence tomography
SD	Spectral domain
SS	Swept source
ED	Enhanced depth
OCTA	OCT angiography
AMD	Age-related macular degeneration
MNV	Macular neovascularization
MH	Macular hole
RPE	Retinal pigment epithelium
RRD	Rhegmatogenous retinal detachment
TRD	Tractional retinal detachment
RS	Retinoschisis
DR	Diabetic retinopathy
NPDR	Non-proliferative diabetic retinopathy
PDR	Proliferative diabetic retinopathy
MAs	Microaneurysms
FA	Fluorescein angiography
UWF	Ultra-widefield
FTMH	Full-thickness MH
DONFL	Dissociated optic nerve fiber layer
ILM	Internal limiting membrane
FAZ	Foveal avascular zone
ERM	Epiretinal membrane
iERM	Idiopathic epiretinal membrane
EIFL	Ectopic inner foveal layer
EZ	Ellipsoid zone
ELM	External limiting membrane
SCP	Superficial capillary plexus
RPC	Radial peripapillary capillary
AI	Artificial intelligence
DRIL	Disorganization of retinal inner layer
BCVA	Best corrected visual acuity
CVI	Choroidal vascular index
IS/OS	Inner segment/outer segment
ICCs	Intraretinal cystic cavities
HRDs	Hyperreflective dots
HRPs	Hyperreflective points
